# A Systematic Review on Biomarkers for Gestational Diabetes Mellitus Detection in Pregnancies Conceived Using Assisted Reproductive Technology: Current Trends and Future Directions

**DOI:** 10.3390/ijms26178234

**Published:** 2025-08-25

**Authors:** Angeliki Gerede, Efthymios Oikonomou, Anastasios Potiris, Christos Chatzakis, Peter Drakakis, Ekaterini Domali, Nikolaos Nikolettos, Sofoklis Stavros

**Affiliations:** 1 Unit of Maternal-Fetal-Medicine, Department of Obstetrics and Gynecology, Medical School, Democritus University of Thrake, GR-68100 Alexandroupolis, Greece; eftoikonomou@outlook.com (E.O.); nnikolet@med.duth.gr (N.N.); 2 Third Department of Obstetrics and Gynecology, Medical School, National and Kapodistrian University of Athens, GR-11527 Athens, Greece; apotiris@med.uoa.gr (A.P.); sfstavrou@med.uoa.gr (S.S.); 3 Second Department of Obstetrics and Gynecology, Medical School, Aristotle University of Thessaloniki, GR-54640 Thessaloniki, Greece; cchatzakis@gmail.com; 4 First Department of Obstetrics and Gynecology, Medical School, National and Kapodistrian University of Athens, GR-11528 Athens, Greece; pdrakakis@hotmail.com (P.D.); kdomali@yahoo.fr (E.D.)

**Keywords:** gestational diabetes mellitus (GDM), assisted reproductive technology (ART), biomarkers, early prediction, IVF pregnancies

## Abstract

Gestational diabetes mellitus (GDM) is a frequently encountered medical complication during pregnancy that is increasing at a rapid pace globally, posing significant public health concerns. Similarly, there is a rising trend in the number of women who have utilized assisted reproductive technology (ART). Numerous studies have been carried out to investigate the relationship between GDM and ART. This comprehensive systematic review seeks to identify potential biomarkers for the early diagnosis of GDM in pregnancies conceived through ART. We conducted a PubMed search covering the past five years to identify studies that explore biomarkers associated with the development of GDM in pregnancies conceived through ART. The outcome measures included human chorionic gonadotropin (HCG), the body mass index (BMI), the Follicle Stimulating Hormone to Luteinizing Hormone (FSH/LH) ratio, increased hemoglobin A1c levels, fasting insulin concentrations, homeostatic model assessment of insulin resistance (HOMA-IR), triglyceride levels, total cholesterol levels, low-density lipoprotein cholesterol concentrations, low-density lipoprotein/high-density lipoprotein (LDL/HDL), total cholesterol to high-density lipoprotein (TC/HDL), the estradiol/follicle ratio, soluble fms-like tyrosine kinase-1 (sFlt-1), Placental Growth Factor (PLGF), endometrial thickness, and psychological stress. Seventeen studies were included. The identification and development of serum or ultrasound biomarkers for the early detection of GDM in pregnancies conceived through ART pose considerable challenges. These challenges arise from the multifactorial nature of GDM, the methodological variations in ART, and the limited availability of relevant studies. The most promising biomarker identified was the estradiol/follicle ratio. Women with a higher estradiol/follicle ratio exhibited significantly lower rates of GDM. There is a pressing necessity for biomarkers to enable the early detection of GDM in pregnancies conceived through ART. E2 levels, β-hCG, and the E2/F ratio, along with the TC/HDL and LDL/HDL ratios, show potential as reliable biomarkers for identifying GDM.

## 1. Introduction

This review aimed to integrate the most reliable evidence available on the availability of biomarkers for assessing the increased likelihood of GDM among singleton gestations resulting from in vitro fertilization (IVF).

The number of pregnancies that happen via assisted reproductive technology (ART) has increased significantly in the last several decades. This is because fertility treatments have improved a lot, and there is a steadily increasing demand for them because of infertility, delayed childbearing, and other reproductive problems. Intracytoplasmic sperm injection (ICSI) and in vitro fertilization (IVF) are the most used procedures. Together, they account for a large number of births globally. ART has helped millions of couples, but new research shows that pregnancies that start this way may be more likely to have bad consequences for the mother and baby, like gestational diabetes mellitus (GDM) [1,2,3].

Gestational diabetes mellitus (GDM) is a transitory kind of diabetes that normally goes away after delivery. It is characterized by an inability to tolerate glucose. It has a lot of repercussions, though, because it makes both women and children more likely to be diagnosed with long-term metabolic disorders, as well as preeclampsia, cesarean delivery, fetal macrosomia, and neonatal hypoglycemia [4]. The number of people with GDM is rising around the world, along with the number of people who are obese and older mothers, which are two important risk factors for the condition. Women who become pregnant through ART often have overlapping risk factors, such as being overweight, being older than 35, or having polycystic ovarian syndrome (PCOS) [5,6,7]. This makes the metabolic landscape of these pregnancies even more difficult.

There has been a lot of research on the possible link between ART and GDM, but the exact reasons why this happens are still not clear. Firstly, the high amounts of hormones such as progesterone and estradiol during ovarian stimulation may change how sensitive insulin is and how quickly glucose is used [8]. Secondly, it is widely supported that adding progesterone, which is often used in ART procedures during luteal phase support, may disrupt the metabolic balance of pregnant women [9]. Also, ART methods like embryo culture and cryopreservation may cause epigenetic changes that affect how the fetus grows and how the placenta works [10]. Finally, abnormal placentation, which happens more often in ART pregnancies, may affect insulin resistance and the pathogenesis of GDM [11].

Even though there are these links, it is still hard for doctors to find GDM early in ART-assisted pregnancies. Interventions that could help reduce bad outcomes are put off since most screening methods, such as oral glucose tolerance testing (OGTT), are performed in the second trimester. Biomarkers that may be found in high-risk women in the first trimester or even before pregnancy would change prenatal care by enabling timely lifestyle modifications, pharmacological interventions, and closer monitoring [12].

This systematic review’s purpose is to gather the most recent information on possible GDM biomarkers in pregnancies that were conceived with ART. We look at serum biomarkers that could show the risk of GDM early in pregnancy, like lipid profiles, hormonal ratios, and inflammatory markers. We also look at ultrasound parameters, like the thickness of the endometrium and the vascularization of the placenta, and new indices, like the estradiol/follicle ratio. By addressing the methodological heterogeneity and gaps in existing studies, this review seeks to (a) point out which biomarkers are most likely to help find GDM early, (b) clarify how ART methods and metabolic results affect each other, and (c) set the stage for future studies that will look at personalized risk assessment and therapy. This research is crucial for both clinical and public health because ART and GDM are becoming more frequent. Finding high-risk pregnancies early on could save medical costs, improve outcomes for mothers and babies, and help obstetricians and ART doctors make better recommendations.

## 2. Materials and Methods

### 2.1. Literature Review

A literature search in PubMed/MEDLINE was conducted independently by two reviewers, covering the past ten years, up to March 2025. The present review adhered to the PRISMA guidelines for systematic reviews and meta-analyses [13]. The systematic review was registered in the International Platform of Registered Systematic Review and Meta-analysis Protocols (INPLASY) with registration number INPLASY202550080 on the 27th of May 2025 and with the DOI 10.37766/inplasy2025.5.0080.

### 2.2. Search Strategy

The inclusion criteria for this systematic review were defined using the PICO framework as follows:

Population—singleton pregnancies conceived via ART.
  Intervention—pregnancies complicated by GDM.
  Comparator—gestational diabetes mellitus (GDM) versus normoglycemic pregnancies.
  Outcome—biomarkers.

A Boolean search was employed using the MeSH terms “gestational diabetes,” “IVF,” and “ART.”

### 2.3. Selection of Studies

Two independent authors screened them for relevance based on titles and abstracts only. Disagreements were resolved through consensus or by discussion with a third author. Articles deemed as irrelevant were excluded, and the full text copies of the remaining articles were assessed for eligibility as per the PICOS criteria by two blinded reviewers. Inconsistencies were, once again, resolved by consensus or by a third reviewer. The references of the full-text copies were accessed to prevent the potential loss of eligible studies that were missed by the database search (snowball procedure). The inclusion criteria were established prior to the commencement of the literature search and were as follows:

(i) Comparative evidence on the incidence of GDM in women who achieved a singleton pregnancy through ART;
(ii) Only full-text, peer-reviewed articles;
(iii) Studies of any design published within the last ten years;
(iv) Articles written in English;
(v) Availability of an abstract.

## 3. Results

**Figure 1:**
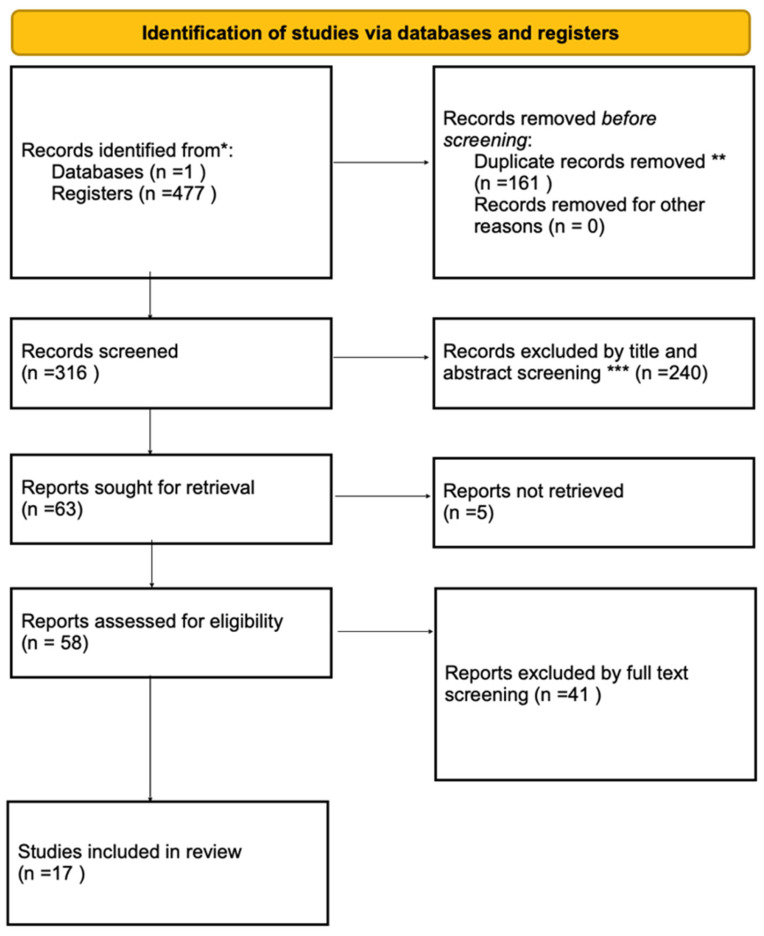
PRISMA 2020 flow diagram for new systematic reviews that included searches of databases and registers only. * Database: PubMed. ** By keywords. *** Study design does not meet the criteria, reviews, there is no comparison group, no human studies.

The initial search identified 464 studies. After the screening process, seventeen studies were included in this systematic review as they fulfilled the eligibility criteria (Figure 1 and Table 1).

### 3.1. BMI

Coussa et al. [14] examined 158 pregnancies conceived through ART, of which 34 developed GDM and 124 did not. The study demonstrated that a significant baseline predictor for the onset of GDM was a higher BMI (29.0 vs. 25.8 kg/m^2^). Also, at 12 weeks, significant predictors of GDM included maternal weight gain (Δ: 3.4 kg vs. 1.5 kg). After adjusting for PCOS and maternal age, BMI at 12 weeks still was a significant predictor [14].

Sun et al. demonstrated that, in pregnancies conceived through ART, GDM is more frequently observed in women with a BMI over 23, regardless of maternal age [23]. Kouhkan et al. found that the odds of developing GDM were increased by 3.27 times in overweight women and by 5.14 times in those classified as obese [24]. Xiong et al. demonstrated that each one-unit increase in body mass index was associated with a 15% higher risk of developing gestational diabetes mellitus [26].

### 3.2. Serum Biomarkers

Kouhkan et al. showed that each 1 mg/dL rise in fasting glucose level was associated with a 17% increase in the risk of developing GDM [24].

Liu et al. demonstrated that multivariate logistic regression analysis, which accounted for age, BMI, and fasting glucose, did not reveal a significant effect of either free or total vitamin D levels on the incidence of GDM (*p* = 0.266 and 0.123, respectively) [25].

Coussa et al. [14] demonstrated that an increased FSH/LH ratio (1.2 compared to 1.0) serves as a significant baseline predictor for the onset of GDM. This study found that key predictors of GDM emergence included a higher BMI (29.0 vs. 25.8 kg/m^2^), advanced maternal age (34 vs. 32 years), increased hemoglobin A1c levels (5.5% vs. 5.2%), higher fasting insulin concentrations (10.6 vs. 7.1 μIU/mL), greater insulin resistance as measured by the HOMA-IR (2.2 vs. 1.7), elevated total cholesterol levels (199 vs. 171 mg/dL), and increased low-density lipoprotein cholesterol (LDL-C) concentrations (123 vs. 105 mg/dL). In contrast, lower triglyceride levels (74 vs. 76 mg/dL) were observed among individuals who later developed GDM [14].

At 12 weeks, important predictors of GDM included increased maternal weight gain (Δ: 3.4 vs. 1.5 kg) along with elevated insulin levels (11.3 vs. 7.6 μIU/mL), triglycerides (178 vs. 120 mg/dL), and HOMA-IR (2.3 vs. 1.5). Following adjustments for PCOS and maternal age, BMI at 12 weeks continued to be a key risk factor for GDM [14].

Shiqiao et al. reported that gestational diabetes mellitus (GDM) occurred most frequently when the estradiol (E2) level per oocyte was below 200 pg/mL [27].

According to Chen et al. [17], pregnant women with initial E2 concentrations above 31.50 pg/mL had a notably lower incidence of GDM at 13.51%, compared to 19.42% in those with lower levels (*p* < 0.01). Similarly, an E2 level greater than 3794.50 pg/mL at the time of hCG injection was associated with a reduced GDM rate of 12.26%, versus 19.32% among those below this threshold (*p* < 0.001). A comparable trend was observed in women whose E2 levels increased by more than 3771.50 pg/mL, where GDM incidence was 12.24%, in contrast to 19.28% in the lower group (*p* < 0.001). A lower E2/F ratio (OR: 1.63, 95% CI: 1.23–2.15, *p* < 0.01), diminished E2 values on the day of hCG trigger (OR: 1.51, 95% CI: 1.13–2.02, *p* = 0.01), and a smaller increase in E2 levels (OR: 1.52, 95% CΙ: 1.14–2.03, *p* < 0.01) were all recognized as significant predictors of heightened GDM risk, after adjusting for confounding variables [17].

Ip et al. found that in pregnancies complicated by GDM, no notable variations in PlGF and sFlt-1 levels were observed when compared to uncomplicated pregnancies following ΙVF/ET [19].

Wu et al. showed that hCG levels were significantly higher in the non-GDM group at 14 or 16 days post-oocyte retrieval. Lower hCG levels may assist as a possible indicator for assessing the risk of developing GDM [15].

Liu et al. [16] indicated that the TC/HDL and LDL/HDL ratios were the strongest predictors of GDM incidence, with odds ratios of 1.96 (95% CI: 1.26–3.04) and 1.942 (95% CI: 1.24–3.03), respectively. Further analysis demonstrated that an increased LDL/HDL ratio heightened the risk of GDM in subgroups typically associated with lower GDM prevalence, including younger women with lower blood pressure and BMI. Lipid ratios showed significant interactions with baseline age, LH, and fasting plasma glucose [16].

Xia et al. [18] found that among singleton live births, the incidence of GDM increased significantly across quartiles of SUA levels, ranging from 5.9% in the lowest quartile to 13.9% in the highest (*p* trend < 0.001). After adjusting for confounding factors, including BMI, blood pressure, blood lipid-related markers and fasting blood glucose, the risk of GDM remained markedly elevated with higher SUA levels [18].

### 3.3. Serum and Ultrasonographic

Chen et al. [17] proposed calculating the E2/F ratio by dividing the peak level of estradiol, measured at the time of hCG injection, by the follicle count present on the same day. On the day of hCG injection, the optimal threshold for the E2/F ratio was determined to be 246.03 pg/mL. Women whose E2/F ratios were above this value experienced a significantly lower incidence of GDM, with a rate of 12.75% compared to 20.41% in those below the threshold (*p* < 0.001) [17]. Furthermore, lower GDM rates were observed in women with baseline estradiol (E2) levels above 31.5 pg/mL (13.5% vs. 19.4%, *p* < 0.01), E2 levels exceeding 3794.50 pg/mL at the time of hCG injection (12.2% vs. 19.3%), and an E2 increase greater than 3771.50 pg/mL (12.2% vs. 19.3%). After adjusting for potential confounders, a lower E2/F ratio (OR: 1.62, 95% CI: 1.22–2.15), reduced E2 levels on the day of hCG administration (OR: 1.51, 95% CI: 1.13–2.02), and a smaller increase in E2 levels (OR: 1.52, 95% CI: 1.14–2.03) were recognized as significant risk factors for GDM [17].

Liu et al., in a study involving 9266 women following fresh in IVF/ICSI-ET cycles, categorized the women into three groups based on endometrial thickness (EMT): 544 women with an EMT of ≤8 mm, 6234 women with an EMT between 8 and 12 mm, and 2488 women with an EMT greater than 12 mm. The comparison did not yield statistically significant results in the rates of GDM among the different groups [21].

Guo et al., in a study involving 3157 participants younger than 42 years who underwent IVF or ICSI treatment followed by fresh ET, classified EMT into three groups: no greater than 7.5 mm, from 7.5 to 12 mm, and greater than 12 mm. Following fresh IVF/ICSI-ET, the data did not indicate an association between endometrial thickness and the risk of GDM [22].

Nguyen-Hoang et al. [29] found that throughout pregnancy, the GDM ± LGA group had a significantly lower estimated marginal mean log10 PlGF multiple of the median (MoM) than the uncomplicated group (−0.01536 versus 0.05572; *p* = 0.032). In contrast, no significant differences were detected between the two groups in the estimated marginal mean log10 MoM values for MAP, UtA-PI, or sFlt-1 during pregnancy [29].

### 3.4. Psychological Stress

Mínguez-Alarcón et al. [20], in a study involving 1324 women, assessed psychological stress prior to conception using the short form of the validated PSS-4. The study found that psychological stress can increase glucose concentrations. The adjusted marginal means (95% CΙ) for mean glucose levels in women within the 1st, 2nd, and 3rd tertiles of psychological stress were 115, 119, and 124 mg/dL, respectively. Additionally, women in the 2nd and 3rd tertiles of psychological stress had a 4% and 13% higher likelihood of demonstrating abnormal glycemic values compared to those in the 1st tertile [20].

### 3.5. Others

Kouhkan et al. [28] demonstrated that injectable progesterone administered during the initial 10 to 12 weeks of gestation was linked to nearly double the likelihood of developing gestational diabetes mellitus (GDM), with an odds ratio (OR) of 2.28 (95% confidence interval CI]: 1.27–4.09), when compared to vaginal progesterone. Additionally, multivariable regression analysis highlighted prior ovarian hyperstimulation syndrome (OHSS) risk (OR: 2.40, 95% CI: 1.34–4.31) and a history of polycystic ovary syndrome (PCOS) (OR: 2.76, 95% CI: 1.26–6.06) as prominent independent risk factors for GDM [28].

Gao et al. [30] reported that, among women who conceived through ART, a sleep duration exceeding 10 h per night was significantly linked to a higher risk of developing GDM. The association between sleep duration and GDM was found to vary with maternal age, with an elevated risk observed only in women under the age of 35. Furthermore, the study did not identify any statistically significant correlation between sleep quality and the occurrence of GDM [30].

### 3.6. Risk of Bias Assessment

A formal quality assessment using the Newcastle–Ottawa Scale was performed for all 17 included studies. The majority were found to have a moderate to low risk of bias. The overall quality of evidence was deemed sufficient to support the review’s conclusions. A full summary of NOS scoring is provided in Table 2.

High-quality studies with a score of 8★ were characterized by prospective designs, large samples and robust adjustment for confounders such as BMI, age, lipid profiles, etc. Moderate-quality studies with a score of 7★ had limitations in terms of comparability (incomplete adjustment for ART protocols), design (potential recall bias associated with retrospective studies), heterogeneity in GDM diagnostic criteria, and a lack of adjustment for lifestyle factors such as diet and exercise. Finally, lower-quality studies with a score of 6★ were characterized by small samples, a lack of confounder adjustment, or/and post hoc analyses.

## 4. Discussion

Multiple studies have shown an increased likelihood of developing GDM in pregnancies resulting from IVF [1,31,32]. This association persisted even after adjusting for causal factors, including a previous occurrence of GDM, a familial predisposition to diabetes and a history of delivering a macrosomic infant—an analytical step frequently overlooked in studies [33]. A possible explanation for the elevated risk of GDM following the use of ART could be the administration of progesterone to support the luteal phase, as well as its continued use throughout the first trimester of pregnancy or for preterm birth prevention [34,35,36]. Conversely, the evidence regarding women who receive vaginal progesterone treatment remains inconclusive [36,37].

The heightened risk is likely the consequence of the fact that women undergoing infertility treatment are often older, making them more susceptible to GDM due to advancing age [38]. However, the relationship between ART and GDM persists even after adjusting for confounding factors. This suggests that ART techniques might induce molecular modifications that increase susceptibility to GDM [8].

IVF has been linked to an elevated risk of unfavorable outcomes such as GDM, even after adjusting for key risk factors. This may be due to hormonal treatments, such as progesterone and elevated estradiol levels during ovarian stimulation, which could impact implantation, placentation, and metabolism. Older maternal age and infertility itself may also contribute to the increased risk [39,40]. Furthermore, it has been proposed that levels exceeding physiological norms of steroid hormones during fresh stimulated cycles may result in abnormal placentation due to the disruption of endometrial angiogenesis [41].

Beyond hormonal alterations, the handling of gametes throughout key IVF procedures—including oocyte retrieval, conventional insemination or ICSI, embryo culture, and ET—might also lead to adverse outcomes. Data suggest that epigenetic modifications, potentially induced by disruptions to the hormonal milieu and the manipulation of gametes and embryos during fundamental developmental windows, may play a role in these effects [42,43]. GDM has been linked to alterations in placental gene regulation, a process thought to be mediated, partially, by epigenetic mechanisms [5,44,45].

Although pregnancies conceived through ART have been associated with a higher incidence of GDM, it is important to note that the increased risk of GDM, detected solely after fresh embryo transfer, may be explained by disparities in placentation quality between fresh and frozen-thawed cycles. These differences are likely explained by variations in the hormonal context of the peri-implantation phase [46,47,48].

There was considerable variability in the definition of GDM across the studies mentioned above. Additionally, significant heterogeneity has been observed in recent efforts to identify biomarkers for GDM. Additionally, there remains a lack of biomarkers for the early detection of GDM in pregnancies achieved through ART.

The most promising biomarker identified in Chen et al.’s study was the E2/follicle ratio. An optimal threshold of 246.03 pg/mL for the E2/F ratio was determined on the day of hCC injection. Women with increased E2/F ratios exhibited markedly lower rates of GDM, with a rate of 12.75% compared to 20.41% in those below the threshold (*p* < 0.001) [17].

Similarly, Shiqiao et al. reported that the incidence of GDM was highest when the E2 concentration per oocyte was less than 200 pg/mL in fresh cycles [27].

In animal models, E2 plays a central role in regulating endometrial development and uteroplacental blood flow, with high concentrations theoretically linked to abnormal placentation. Recent studies have shown that elevated E2 levels may disrupt the expression of implantation-related genes, potentially contributing to defective placental formation [49]. In obese mouse models, E2 has been shown to mitigate metabolic disturbances linked to GDM. Post-pregnancy, these mice exhibited improved glucose tolerance, likely due to E2’s role in enhancing insulin secretion and regulating hepatic glucose metabolism through the activation of the AKT pathway and increased cAMP signaling [50]. Intervention with externally administered sex steroids, along with GnRH and gonadotropins, has the potential to disturb the regulatory mechanisms of the hypothalamic–pituitary–gonadal axis. Such disturbances may give rise to atypical endocrine profiles, which have been implicated in an increased susceptibility to GDM [51]. As E2 is produced by granulosa cells, its concentration may serve as an indirect marker of oocyte quality. In the context of ART, E2 levels during fresh cycles have been shown to correlate positively with the number of oocytes retrieved [27].

Some studies demonstrated that BMI and weight gain can serve as predictors of GDM in pregnancies achieved through ART [14,23,24,26].

Identifying and developing serum or ultrasound biomarkers for the early detection of GDM in pregnancies achieved through ART presents significant challenges. One of the main difficulties lies in the complex and multifactorial nature of GDM, which involves a range of genetic, hormonal, and environmental factors. The hormonal treatments used in ART, such as gonadotropins and progesterone, can further complicate the identification of biomarkers by altering the metabolic and endocrine environment in ways that may not be seen in natural pregnancies.

Additionally, variations in the timing and methodology of ART procedures add to the complexity of finding reliable biomarkers. Given the increasing prevalence of ARTs and their association with adverse pregnancy outcomes, it is essential to classify ARTs according to their specific protocols and cycle types when investigating complications such as GDM. Distinguishing between fresh and frozen ET procedures allows for a more accurate assessment of metabolic outcomes, as hormonal milieu and endometrial preparation differ substantially between the two. For instance, frozen cycles often involve artificially prepared endometria, potentially lacking the physiological hormonal fluctuations seen in natural or stimulated fresh cycles. Such variations may influence maternal glucose metabolism and placental development, thereby affecting GDM risk.

In addition to cycle type, ART protocols vary according to the use of GnRH agonists or antagonists, each exerting distinct effects on ovarian stimulation and systemic hormonal levels. Differing endocrine profiles may have implications for early placental development and insulin sensitivity, both of which are critical factors in GDM pathophysiology. Thus, stratifying ARTs by both embryo transfer type and stimulation protocol is vital to elucidating their differential impact on metabolic pregnancy outcomes.

Accounting for these distinctions also has implications for clinical practice. As personalized medicine continues to evolve, tailoring ART protocols to minimize metabolic risk becomes increasingly relevant. By systematically categorizing ART treatments according to stimulation method and embryo transfer type, clinicians and researchers can better identify subgroups of patients who are at elevated risk of developing GDM.

The heterogeneity in the patient population, including factors such as age, underlying fertility issues, and prior pregnancy history, also contributes to the difficulty in developing universally applicable biomarkers. Moreover, the dynamic changes in metabolic and hormonal factors during the early weeks of gestation further challenge the identification of biomarkers that are both specific and sensitive for early GDM detection.

Future research examining complications related to ART should employ a stratified analytical framework that considers both the type of embryo transfer cycle—whether frozen or fresh—and the specific ovarian stimulation protocol utilized, such as GnRH agonist or antagonist regimens. This approach would enhance the granularity and clinical relevance of findings, enable a more accurate identification of risk patterns, and facilitate targeted preventative strategies for conditions such as GDM.

## 5. Conclusions

There is a critical need for biomarkers to facilitate the early detection of GDM in pregnancies achieved through ART. Early identification of GDM is essential for managing the condition and preventing adverse outcomes for both mother and fetus. Given that pregnancies resulting from ART are linked to an elevated risk of GDM, developing reliable biomarkers would allow for timely interventions. It appears that levels of Ε2, β-hCG, the E2/F ratio, as well as the TC/HDL and LDL/HDL ratios, are promising biomarkers for the detection of GDM. To verify the reliability of these and other potential biomarkers, a multi-center prospective cohort study is recommended, which has not yet been conducted. This study should enroll at least 2,000 women undergoing ART, stratify by ART protocol (fresh vs. frozen embryo transfer, GnRH agonist vs. antagonist), and assess a comprehensive panel of serum (e.g., E2, β-hCG, TC/HDL, LDL/HDL, SUA) and ultrasonographic (e.g., EMT, placental volume, VI, FI) biomarkers in the first trimester or pre-pregnancy. Such a design would address the methodological heterogeneity of ART, account for patient variability (e.g., BMI, PCOS) establishing predictive thresholds for early GDM detection that will enable personalized risk assessment and improved clinical outcomes. The identification of effective biomarkers is crucial for better outcomes in ART pregnancies.

## Figures and Tables

**Table 1:** Studies incorporated into the final analysis.

Author, Year,	Sample	Study Design	Biomarkers	Method	Outcome
Coussa et al., 2021 [14]	158	Prospective cohort study	Preconceptional BMI, age, FSH/LH ratio, hemoglobin A1c, insulin, HOMA-I, TC, LDL, Triglycerides, maternal weight gain	The study participants comprised a 28-week prospective cohort of pregnant women who achieved pregnancy through in vitro fertilization (IVF). Eligible participants were aged ≤39 years, had a body mass index (BMI) ranging from 18.5 to 38 kg/m^2^, and had no prior diagnosis of DM. Fasting blood samples were obtained prior to IVF treatment and again at 12 weeks of gestation to evaluate thyroid function, reproductive hormones, glucose levels, serum insulin, lipid profiles, adiponectin, and concentrations of lipopolysaccharide-binding protein.	GDM was diagnosed in 34 women, whereas 124 did not develop the condition. Key baseline predictors associated with the development of GDM included an elevated BMI, increased maternal age, an elevated FSH/LH ratio, increased hemoglobin A1c levels, elevated total cholesterol levels, higher fasting insulin concentrations, greater insulin resistance as assessed by the HOMA-I, and increased low-density lipoprotein cholesterol concentrations. Conversely, lower triglyceride levels were observed among those who developed GDM. At 12 weeks, notable indicators of GDM included greater maternal weight gain and higher concentrations of insulin, triglycerides, and HOMA-IR. After adjusting for PCOS status and maternal age, BMI at 12 weeks remained an independent and key determinant of GDM.
Wu et al., 2022 [15]	1005	Retrospective cohort analysis	hCG levels in early pregnancy	Medical records were reviewed for women who underwent either fresh or frozen embryo transfer (ET) and achieved a live birth. The period of study was from October 2015 to June 2020. The analysis included autologous in IVF or ICSI cycles, in which 1 or 2 embryos were transferred on either day 2, 3, or 5.	This study demonstrated that initial hCG levels were higher in the non-GDM group at 14 or 16 days after oocyte retrieval. Lower hCG levels in early pregnancy may function as a possible indicator for assessing the risk of GDM in later stages of gestation.
Liu et al., 2024 [16]	767	Retrospective study	Age, BMI, LDL, TC, and TG, and lower HDL as well as pre-pregnancy LDL/HDL and TC/HDL	Data collection took place in Changsha, China, at the Reproductive and Genetic Hospital of CITIC-Xiangya over a two-year period (2017 to 2018). The lipid profile assessments comprised measurements of LDL, HDL, TC, and TG.	A total of 119 individuals developed GDM, whereas 648 did not. LDL and HDL were strongly correlated with GDM, whereas TC and TG did not show such associations. The TC/HDL and LDL/HDL ratios were strongly linked to GDM. Further analysis revealed that a higher LDL-to-HDL ratio increased the risk of GDM in subgroups typically associated with a lower GDM prevalence.
Chen et al., 2024 [17]	1593	A Post hoc analysis of a prospective cohort study	E2/F ratio on the day of hCG administration	Participants were required to be between the ages of 18 and 39 years. Additionally, they had to be undergoing their first cycle of IVF or ICSI. Only those who received a fresh embryo transfer and subsequently became pregnant, as confirmed by ultrasound, were included. All participants underwent ovarian stimulation using an agonist protocol. The E2/F ratio is calculated by dividing the peak estradiol level (measured on the hCG day) by the follicle count.	An optimal threshold for the E2/F ratio at the time of hCG administration was identified as 246.03 pg/mL. Women whose E2/F ratio exceeded this value demonstrated lower rates of GDM, at 12.75%, compared to 20.41% among those below the threshold (*p* < 0.001). Reduced incidences of GDM were also noted in women with baseline Ε2 levels above 31.50 pg/mL, Ε2 levels at the time of hCG administration exceeding 3794.50 pg/mL, and an E2 elevation greater than 3771.50 pg/mL. Conversely, a lower E2/F ratio, reduced E2 concentrations at the time of hCG administration, and a smaller increment in E2 levels were classified as substantial risk factors for GDM.
Xia et al., 2024 [18]	13,325 without PCOS	Retrospective study	SUA levels	This study, conducted retrospectively in China at a university-linked reproductive medicine center, involved women who underwent their 1st fresh IVF or ICSI embryo transfer cycles, between January 2014 and December 2022. It aimed to explore patterns in obstetric, perinatal outcomes and in pregnancy across the different quartiles of SUA levels.	The proportion of women developing GDM rose significantly from the first to the fourth quartile of SUA levels (5.9% to 13.9%, Ptrend < 0.001). After adjusting for potential confounders, including fasting blood glucose, BMI, blood pressure and lipid-related indicators, the incidence of GDM demonstrated a notable increase.
Ip et al., 2024 [19]	123	Prospective cohort study	MAP, VI, FI, VFI, mUtPI, PIGF, sFlt-1	A prospective cohort study was carried out from December 2017 to January 2020. At the time of the nuchal translucency ultrasound examination, MAP, placental volume, VI, FI, VFI, mUtPI and biochemical markers like PlGF and sFlt-1 were assessed.	Pregnancies complicated by GDM showed no significant differences in PlGF and sFlt-1 levels when compared to uncomplicated pregnancies following IVF/ET.
Mínguez-Alarcón et al., 2024 [20]	1324	Prospective cohort study	Psychological Stress based on PSS-4	Prior to conception, participants completed a psychological stress questionnaire based on Perceived Stress Scale 4 (PSS-4). During the later stages of pregnancy, blood glucose levels were assessed with a 50 g glucose drink. To explore the relationship between total PSS-4 scores and both mean glucose levels and the incidence of elevated glucose (≥140 mg/dL), log-binomial regression and linear models were utilized.	An association between psychological stress and mean abnormal glucose levels was observed. Furthermore, women in the 2nd and 3rd tertiles of psychological stress were 4% and 13% more likely to exhibit abnormal glucose levels when contrasted with those in the 1st tertile (*p* for trend = 0.01).
Liu et al., 2021 [21]	9266	Retrospective cohort study	EMT	This retrospective cohort study encompassed women who experienced singleton live births after undergoing fresh IVF/ICSI-ET cycles at the Center for Reproductive Medicine, affiliated with Shandong University. The period of study was from January 2014 to December 2018.	Among the different groups categorized according to EMT, no statistically significant differences in GDM percentages were observed.
Guo et al., 2020 [22]	3157	Retrospective cohort study	EMT	Focusing on women under the age of 42, this retrospective cohort study included those who underwent IVF or ICSI treatment followed by fresh ET at the Reproductive Hospital of Shandong University, China, all of whom had live singleton births. The period of study was between January 2017 and December 2018.	Endometrial thickness was unassociated with the risk of GDM.
Sun et al., 2022 [23]	3043	Retrospective cohort study	Pre-pregnancy BMI	Retrospective cohort study in Shanghai First Maternity and Infant Hospital, between January 2015 and August 2020.	Women who were overweight or obese prior to conceiving singleton pregnancies via ART demonstrated a higher likelihood of developing gestational diabetes.
Kouhkan et al., 2019 [24]	270	Nested case–control study	Pre-pregnancy BMI, FBS, BMI+FBS	Nested case–control study conducted from October 2016 to June 2017. BMI was categorized according to the criteria established by the World Health Organization (WHO).	Pre-pregnancy BMI and first-trimester FBS independently predict the likelihood of developing GDM in women who conceived through ART. The combined presence of elevated FBS and obesity significantly amplifies the risk of GDM in this population.
Liu et al., 2023 [25]	1593	Post hoc analysis of a prospective study	Pre-pregnancy total and free vitamin D	Post hoc analysis of a prospective study at the Reproductive and Genetic Hospital of CITIC-Xiangya in Changsha, China	The occurrence of GDM was not linked to the severity of either total or free vitamin D deficiency before pregnancy.
Xiong et al., 2022 [26]	6598	Population-based retrospective cohort study	maternal pre-pregnancy bodyweight	This population-based retrospective cohort study, conducted in China, analyzed pregnancies resulting from ART recorded in a pregnancy registration database between January 2014 and March 2019.	A linear dose–response association exists between pre-pregnancy body weight and the risk of GDM after ART treatment.
Shiqiao et al., 2020 [27]	1022	Retrospective cohort study	Basal FSH, AFC, infertility years, gestational age, number of fetus, method of fertilization, and reason of infertility, age, BMI, and fresh cycle, E2 level	A retrospective cohort study from 1 January 2014, to 31 August 2017.	The incidence of GDM was highest when the E2 level was below 200 pg/mL per oocyte.
Kouhkan et al., 2018 [28]	270	Nested case–control study	The route of progesterone administration, previous OHSS risk and history of PCOS	A nested case–control study was conducted between October 2016 and June 2017.	Potential risk factors for GDM among women who conceived via ART include the method of progesterone administration, a previous risk of OHSS, and a history of PCOS.
Nguyen-Hoang et al., 2024 [29]	143	Prospective longitudinal study	MAP, UtA-PI, PlGF, sFlt-1	A study was undertaken at the Department of Obstetrics and Gynecology, Prince of Wales Hospital, The Chinese University of Hong Kong, Hong Kong SAR, between December 2017 and January 2020.	GDM is associated with reduced PlGF levels during the latter half of pregnancy.
Gao et al., 2024 [30]	856	Prospective birth cohort study	Sleep Quality using PSQI	In study, sleep characteristics of women who conceived through ART were evaluated in early pregnancy using the Pittsburgh Sleep Quality Index (PSQI).	Sleep quality was not found to be significantly associated with the risk of developing GDM.

**Table 2:** Risk of bias assessment using Newcastle–Ottawa Scale (NOS) of all included studies.

Study (Author, Year)	Selection (Max 4★)	Comparability (Max 2★)	Outcome (Max 3★)	Total Score (Max 9★)	Key Justifications
Coussa et al., 2021 [14]	★★★★	★★	★★★	**8**	Prospective cohort; adjusted for PCOS/age; complete follow-up.
Wu et al., 2022 [15]	★★★★	★	★★★	**7**	Retrospective; controlled for embryo transfer type but not BMI/lifestyle.
Liu et al., 2024 [16]	★★★★	★★	★★★	**8**	Large sample; adjusted for lipid ratios/age/BMI; robust GDM diagnosis.
Chen et al., 2024 [17]	★★★★	★★	★★★	**8**	Defined E2/F ratio threshold; adjusted for confounders; clear outcome.
Xia et al., 2024 [18]	★★★★	★★	★★★	**8**	Large retrospective cohort; adjusted for SUA/BMI/glucose.
Ip et al., 2024 [19]	★★★★	★	★★★	**7**	Controlled placental markers but limited confounder adjustment.
Mínguez-Alarcón et al., 2024 [20]	★★★★	★	★★★	**7**	Measured stress pre-conception; lacked adjustment for ART protocols.
Liu et al., 2021 [21]	★★★★	★	★★★	**7**	Large sample; EMT analysis but no adjustment for hormonal factors.
Guo et al., 2020 [22]	★★★★	★	★★★	**7**	Focused on EMT; minimal control for metabolic confounders.
Sun et al., 2022 [23]	★★★	★	★★★	**6**	Retrospective; adjusted for BMI but not other confounders.
Kouhkan et al., 2019 [24]	★★★	★★	★★★	**7**	Nested case–control; adjusted for BMI/fasting glucose.
Liu et al., 2023 [25]	★★★	★	★★★	**6**	Post hoc analysis; no adjustment for key confounders.
Xiong et al., 2022 [26]	★★★★	★★	★★★	**8**	Population-based; adjusted for pre-pregnancy weight.
Shiqiao et al., 2020 [27]	★★★	★	★★★	**6**	Retrospective; limited confounder adjustment.
Kouhkan et al., 2018 [28]	★★★	★★	★★★	**7**	Adjusted for progesterone/PCOS; nested design.
Nguyen-Hoang et al., 2024 [29]	★★★★	★	★★★	**7**	Prospective; controlled PlGF but limited other adjustments.
Gao et al., 2024 [30]	★★★	★	★★★	**6**	Adjusted for sleep duration but not ART protocols.

★: minimal quality in the respective category (suggesting a high risk of bias), ★★: moderate quality in the respective category (suggesting a moderate risk of bias), ★★★: good quality in the respective category (suggesting a low to moderate risk of bias), ★★★★: high quality in the respective category (suggesting a low risk of bias)

## Data Availability

This study did not create or analyze new data, and data sharing does not apply to this article.

## Abbreviations

The following abbreviations are used in this manuscript:

ART	Assisted Reproductive Technology
BMI	Body Mass Index
E2/F	Estradiol/follicle
EMT	Endometrial thickness
ET	Embryo Transfer
FI	Flow index
GDM	Gestational Diabetes Mellitus
HCG	Human Chorionic Gonadotropin
HDL	High-density lipoprotein
HOMA-IR	Homeostatic model assessment of insulin resistance ICSI-intracytoplasmic sperm injection
IVF	In Vitro Fertilization
LDL	Low-density lipoprotein
mUtPI	Mean uterine artery pulsatility index
PCOS	Polycystic ovary syndrome
PlGF	Placental Growth Factor
PSS-4	Perceived Stress Scale 4
sFlt-1	Soluble fms-like tyrosine kinase-1
SUA	Serum uric acid
TC	Total cholesterol
TG	Triglycerides
VFI	Vascularization flow index
VI	Vascularization index
